# Diagnostic Task Specific Activations in Functional MRI and Aberrant Connectivity of Insula with Middle Frontal Gyrus Can Inform the Differential Diagnosis of Psychosis

**DOI:** 10.3390/diagnostics11010095

**Published:** 2021-01-08

**Authors:** Drozdstoy Stoyanov, Katrin Aryutova, Sevdalina Kandilarova, Rositsa Paunova, Zlatoslav Arabadzhiev, Anna Todeva-Radneva, Stefan Kostianev, Stefan Borgwardt

**Affiliations:** 1 Department of Psychiatry and Medical Psychology, and Research Institute, Medical University Plovdiv, 4002 Plovdiv, Bulgaria; katrin.aryutova@phd.mu-plovdiv.bg (K.A.); sevdalina.kandilarova@mu-plovdiv.bg (S.K.); rositsa.paunova@mu-plovdiv.bg (R.P.); z.arabadzhiev@mu-plovdiv.bg (Z.A.); anna.todeva@mu-plovdiv.bg (A.T.-R.); 2 Department of Pathophysiology, and Research Institute, Medical University Plovdiv, 4002 Plovdiv, Bulgaria; skostianev@pathophysiology.info; 3 Klinik für Psychiatrie und Psychotherapie, Universität zu Lübeck, 23538 Lübeck, Germany; stefan.borgwardt@uksh.de; 4 Department of Psychiatry, University of Basel, 4001 Basel, Switzerland

**Keywords:** neuropsychiatric disorders, translational neuroscience, neuroimaging, brain networks, connectivity, schizophrenia, depression, precuneus, insula, frontal cortex, default mode network

## Abstract

We constructed a novel design integrating the administration of a clinical self-assessment scale with simultaneous acquisition of functional Magnetic Resonance Imaging (fMRI), aiming at cross-validation between psychopathology evaluation and neuroimaging techniques. We hypothesized that areas demonstrating differential activation in two groups of patients (the first group exhibiting paranoid delusions in the context of paranoid schizophrenia—SCH—and second group with a depressive episode in the context of major depressive disorder or bipolar disorder—DEP) will have distinct connectivity patterns and structural differences. Fifty-one patients with SCH (*n* = 25) or DEP (*n* = 26) were scanned with three different MRI sequences: a structural and two functional sequences—resting-state and task-related fMRI (the stimuli represent items from a paranoid-depressive self-evaluation scale). While no significant differences were found in gray matter volumes, we were able to discriminate between the two clinical entities by identifying two significant clusters of activations in the SCH group—the left Precuneus (PreCu) extending to the left Posterior Cingulate Cortex (PCC) and the right Angular Gyrus (AG). Additionally, the effective connectivity of the middle frontal gyrus (MFG), a part of the Dorsolateral Prefrontal Cortex (DLPFC) to the Anterior Insula (AI), demonstrated a significant difference between the two groups with inhibitory connection demonstrated only in SCH. The observed activations of PreCu, PCC, and AG (involved in the Default Mode Network DMN) might be indirect evidence of the inhibitory connection from the DLPFC to AI, interfering with the balancing function of the insula as the dynamic switch in the DMN. The findings of our current study might suggest that the connectivity from DLPFC to the anterior insula can be interpreted as evidence for the presence of an aberrant network that leads to behavioral abnormalities, the manifestation of which depends on the direction of influence. The reduced effective connectivity from the AI to the DLPFC is manifested as depressive symptoms, and the inhibitory effect from the DLPFC to the AI is reflected in the paranoid symptoms of schizophrenia.

## 1. Introduction

Mental disorders constitute one of the major sources of economic burden in modern society in addition to the stigmatization of mental illness. Depressive disorders (DEP) and schizophrenia (SCH), in particular, are accountable for a significant percentage of the disability-adjusted life years as a criterion for economic and social burden worldwide [1]. Furthermore, these disorders, especially the treatment-resistant forms, are associated with highly decreased quality of life and a significant increase in comorbidities and suicide risk [2,3]. Several studies have provided evidence for existing shared genetic correlations between psychiatric conditions such as psychosis, depression, autism spectrum disorder, and bipolar disorder [4,5]. In addition, specific structural abnormalities of the Central Nervous System (CNS) discovered by MRI, such as grey-matter volume reduction in the anterior cingulate cortex (ACC), can define a genetic vulnerability to psychosis [6]. Moreover, a genome-wide association study established that genetic liability for Major Depressive Disorder (MDD) may be related to an increase in suicidality in psychiatric disorders [7]. Unfortunately, this contributes to a growing tendency for stigmatizing attitudes towards schizophrenic patients not only in society as a whole but also among medical professionals [8].

One of the most common debates in psychiatry appears to be related to the fact that diagnosis and treatment decisions rely mainly on patient reports, behavioral observation, and the willingness to make judgments about the underlying inner nature of the patient’s experience, rather than to observe accurate objective biomarkers. The field of psychiatry needs to incorporate a trans-disciplinary approach towards the diagnosis in order to establish a biological cross-validation of clinical phenomenology, which has been missing since its differentiation as an independent medical specialty.

The current diagnostic systems in psychiatry (such as the International Classification of Diseases (ICD) [9] and the Diagnostic and Statistical Manual (DSM) of Mental Disorders [10] are widely used among clinicians, although they have low validity [11]. This is evident from the fact that the diagnostic process is hampered by certain obstacles such as heterogeneity, comorbidity, and unclear distinctions between normal and pathological behavior. Another important issue is that mental disorders are classified based exclusively on clinical characteristics without considering the etiological factors [12]. Translational neuroscience may be the key to establishing fundamental knowledge about the origin of behavioral disorders by identifying and explaining the underlying neurobiological correlates of psychiatric conditions. Neuroimaging techniques, such as functional Magnetic Resonance Imaging (fMRI), offer the possibility for translation between basic neuroscience and pathological behavioral manifestations that correlate with it. The integration of neuroimaging methods and the hitherto acquired know-how of the underlying genetic causes, neurochemical dysfunctions, and neuroinflammatory mechanisms may finally allow a change of the current status of psychiatry [13] and the establishment of evidence-based explanations of the etiology of mental disorders.

In recent decades, many studies have been conducted with the aim to detect structural and functional abnormalities in psychiatric disorders. Despite years of efforts in this area, however, the results remain inconsistent [14]. This may be partly due to the specificity of the design of the methods used. A common practice in fMRI studies is to conduct a pre- and post-scan clinical assessment. This causes a time difference between the two measurements. In certain situations, this could affect the accuracy of the findings (e.g., in bipolar patients with rapid cycling) [15]. To address this issue, it could be beneficial to create or transform some of the current assessment or self-assessment questionnaires via cross-validation against certain neurobiological biomarkers. fMRI could serve as the appropriate support element for an evidence-based design, where the gathered neuroimaging data, together with the parallel implementation of a self-assessment scale [16], could construct a scientifically valid instrument that can be used by clinicians in daily practice with trust in the reliability of the method. Therefore, when using this translational approach, which incorporates fundamental neuroscience, neuroimaging technologies, psychometric instruments, and psychopathology, physicians can rely on an accessible and valid diagnostic tool [17]. The initiative might have an effect not only on the diagnostics, but also on the prevention, treatment, and monitoring of the therapeutic effect, as well as the decision regarding the choice of medications.

As a scientific effort on the application of the translational model in psychiatry, our research group has performed several experiments. We designed a novel paradigm, which integrates a clinical self-assessment scale administered simultaneously with the acquisition of fMRI, aiming at cross-validation between psychopathology evaluation and neuroimaging techniques [12,15,18]. Our research has been conducted in three phases. The first study was designed to integrate the clinical self-assessment scale of von Zerssen [19] administered concurrently with fMRI. We used two conditions during the first phase of the research: diagnostically specific (DS) items applying the depression scale and diagnostically neutral (DN) applying the scale of general interests performed in a block design, contrasting the results between patients suffering from a depressive episode and healthy controls. Thus, we were able to detect distinct activations in the depressed group while processing the DS items that were not present in the control group, thus demonstrating the sensitivity of the test [15]. During the second phase of the research, we upgraded the paradigm by including one more condition (namely the paranoia items from the paranoid–depressive scale—PDS) and we recruited patients with paranoid syndrome in the context of schizophrenia to explore the comparison between the different nosological groups (e.g., the specificity of the test). However, no residual activations were produced in the direct comparison between the two patient groups, which led us to the next level of our experiments.

At the final stage, we applied multivariate analysis to the same dataset, the goal being to implement an unsupervised machine learning approach, where the brain signatures identified would correlate to the different conditions used in the design. By using a multivariate linear model (MLM) and principal component analysis, we were able to differentiate the two psychiatric groups—SCH and DEP. Three brain patterns were established following the individual and group MLM, summarizing all the individual variability of the individual brain patterns. The aforementioned objective of establishing a translationally valid tool in the diagnostic process of schizophrenia and affective disorders is supported by this finding.

In this context, the aim of the present study was to advance the translational approach used so far by combining data already acquired from different modalities, namely high-resolution structural images, resting state, and task-related data. Since in our pilot study the sample size was relatively small, which might have led to the negative results of the direct comparison between the groups when stringent criteria for statistical significance were applied, we hypothesized that increasing the sample would enable us to overcome this issue. In addition, our goal was to explore whether the differences in the brain activations during the task can be translated into or explained by some structural or connectivity changes as well. In order to achieve this, we used voxel-based morphometry analysis to assess the gray matter volumes and spectral dynamic causal modeling to derive the effective connectivity measures of eight specific regions of interest. We hypothesized that the areas demonstrating differential activation in the two groups will simultaneously have distinct connectivity patterns and structural differences.

## 2. Materials and Methods

### 2.1. Participants

For the current study, we recruited 51 patients with a current psychotic episode—in the context of schizophrenia (*n* = 25, mean age 38.8 ± 13.5 y, 13 males), depressive episode (*n* = 26, mean age 41 ± 11.4 y, 9 males), major depressive disorder (*n* = 10, mean age 37.5 ± 9.9 y, 4 males), or bipolar disorder (*n* = 16, mean age 43.1 ± 12.1 y, 5 males)—according to the diagnostic criteria of DSM IV TR [20]. The assessment of the participants was performed by experienced psychiatrists (D.S., S.K., K. A.) using the general clinical interview [21] and the structured Mini International Neuropsychiatric Interview (M.I.N.I 6.0) [22] and Clinical Global Impression (CGI) scale [23] as well as the Montgomery–Åsberg Depression Rating Scale (MADRS) [24] and the Positive and Negative Syndrome Scale (PANSS) [25]. Diagnosis was established based on the clinical interview, the presented medical documentation, and the additional information from accompanying family members (in some cases).

The Bulgarian Translation of the short version of M.I.N.I 6.0 was used in order to confirm the current episode (major depressive or psychotic) and the diagnosis (major depressive disorder, bipolar disorder, or schizophrenia). The same instrument served to rule out comorbid disorders such as panic disorder, agoraphobia, social phobia, generalized anxiety disorder, obsessive-compulsive disorder, post-traumatic stress disorder, eating disorders (anorexia and bulimia), alcohol or other substance use disorders, and dissocial personality disorder. The severity of the depressive episode was further assessed by the use of the MADRS, which is a 10-item clinician-rated scale broadly used both in practical and research settings. We have chosen a cut-off value for the total score of 20, above which depression is generally considered to be at least of moderate severity (severe depression is accepted above 35). The psychotic episode, on the other hand, was additionally evaluated using the PANSS instrument, which allows for detailed scoring of different positive, negative, and general symptoms. To secure a reasonable severity of the episode, we have set a minimum rating of 3 on P1 (delusions) and/or P6 (suspiciousness). Both clinical groups were on stable medication during the past 14 days.

The exclusion criteria were the following: age under the age of 18 or over the age of 65, presence of metal implants or body grafts (e.g., pacemaker) incompatible with MRI, comorbid mental disorder as identified by the clinical interview and the M.I.N.I., (e.g., substance or alcohol use disorder, obsessive-compulsive disorder), severe somatic or neurological disease, and traumatic brain injury with loss of consciousness. Anamnesis of previous episodes and treatments were further considered as a source of information in order to supplement the exclusion criteria. The exclusion criteria used were referenced according to DSM IV TR [26]. Each of the patients provided a written informed consent complying with the Declaration of Helsinki. The protocol of the study was approved by the University’s Ethics Committee.

### 2.2. Image Acquisition

The participants were scanned on a 3T MRI system (GE Discovery 750w) with 3 different MRI sequences: high-resolution structural scan (Sag 3D T1 FSPGR sequence), with slice thickness 1 mm, matrix 256 × 256, TR (relaxation time) 7.2 ms, TE (echo time) 2.3, and flip angle 12°; and two functional scans (2D EPI sequence) while resting with eyes closed—slice thickness 3 mm, 36 slices, matrix 64 × 64, TR, 2000 ms, TE, 30 ms, flip angle 90°, 192 volumes and during the task (see the following paragraph); and slice thickness 3 mm, matrix 64 × 64, TR 2000 ms, TE 30 ms, and flip angle 90°, 256 volumes. The functional scan started with 5 dummy time series, which were automatically excluded.

### 2.3. fMRI Task

The paradigm was created using E-prime software (Psychology Software Tools, Inc., Pittsburgh, PA, USA) and consisted of 32 s blocks with three different active conditions and one 20 s block with the rest condition (fixation cross). The stimuli were presented using Nordic Neuro Lab Visual System. As it is described in detail in our previous work [18,27], we will here briefly summarize it.

The active blocks represented four written statements of 8 s each taken from the von Zerssen paranoia-depression scale. There were Depression Specific (DS) blocks with the statements from the depression subscale (“I often feel simply miserable”, “I don’t have any feelings anymore”) and Paranoid-Specific (PS) blocks from the paranoia subscale (“Other people constantly follow and control me”). The Diagnostically Neutral (DN) blocks included statements from a questionnaire about general interests and likes (such as “I like to write books or plays”, “I like to repair household appliances”, etc.). Four possible answers (“completely true”, “mostly true”, “somewhat true”, “not true”) and the respective four response buttons (upper left, lower left, lower right, upper right) were presented under each statement. The whole task incorporated four blocks of each type, alternating between the three active conditions, followed by the rest condition (DS__rest__DN__rest__PS__rest…). The participants were instructed to read the statements carefully and to respond with a button press according to their level of agreement. During the rest condition, they had to focus on the fixation cross without thinking of anything.

### 2.4. MRI Data Analysis

#### 2.4.1. Structural Data Analysis—Voxel-Based Morphometry (VBM)

The analysis of the MRI images was performed using the SPM 12 (Statistical Parametric Mapping, http://www.fil.ion.ucl.ac.uk/spm/) software running on MATLAB R2020 for Windows and the CAT 12 toolbox implemented in SPM (http://www.neuro.uni-jena.de/software/). The preprocessing of the T1 images encompassed, first, segmentation with the CAT 12 toolbox, including normalization to standardized MNI (Montreal Neurological Institute) space; and second, spatial smoothing with an 8 mm full-width-at-half-maximum (FWHM) Gaussian kernel. In addition, the total intracranial volume (TIV) was calculated for each subject. In the next step, a general-linear model was defined with age, sex, and TIV as covariates. We then compared the grey-matter volumes of the two groups with a two-sample *t*-test. The statistical threshold was set to *p* < 0.05 FWE (Family Wise Error) corrected.

#### 2.4.2. Task-Related Functional Data Analysis

The functional images (both from the task and from the resting state scans) were first realigned for correction of head motion, co-registered with the high-resolution anatomical image, normalized to MNI space, and spatially smoothed with an 8 mm FWHM Gaussian kernel.

Following the preprocessing, a first-level analysis was conducted using a general linear model (GLM) applied to the time series, convolved with a canonical hemodynamic response function. Covariates of no interest included the six rigid body motion correction parameters. Individual T-contrasts were defined for active vs. passive conditions. The contrast maps obtained from each comparison were included in a second-level random-effects analysis to test for differences between the two patient groups (schizophrenia > depression = SCH > D and depression > schizophrenia = D > SCH). The level of significance was set to *p* < 0.05 FWE corrected using an uncorrected cluster-forming threshold of *p* < 0.001. The effects of age and sex were controlled for as they were added as covariates of no interest in the design matrix.

#### 2.4.3. Resting State Data Analysis—Effective Connectivity

Following the preprocessing (same as for the task-related data), first-level resting-state analysis was conducted using a general linear model (GLM) applied to the time series. Nuisance covariates included the six rigid body motion parameters, average white matter, and cerebrospinal fluid signal time series. BOLD time-series were extracted for eight predefined regions of interest of 6 mm radius spheres (3 mm radius for angular gyrus and planum temporale). These were the following left hemisphere regions with their MNI coordinates: precuneus (PreCu) [−10, −64, 24], hippocampus (HPC) [−24, −11, −18], anterior insula (AI) [−34, 22, 4], angular gyrus (AngG) [−26, −80, 42], orbitofrontal cortex (OFC) [−40, 27, −8], planum temporale (PlT) [−54, −33, 15], thalamus (anterior nuclei) (Th) [−6, −10, 2], middle frontal gyrus (MFG) [−41, 19, 41]. BOLD signal from some of the ROIs (OFC) was lacking in one patient which led to the exclusion of this dataset from further analysis.

Spectral dynamic causal modeling (spDCM) was performed with these eight regions of interest. We used a fully connected model where each node was connected to each other node. Further, the individual spDCM models were jointly estimated, using the Parametric Empirical Bayes (PEB) framework, implemented in SPM12. Finally, connectivity strengths (A-matrix) were extracted from the estimated spDCM models, and further statistical analysis in SPSS was performed.

### 2.5. Statistical Analysis

Statistical analysis of the demographic and clinical characteristics of the participants as well as of the connectivity strengths of the spDCM model were performed by means of SPSS 22.0 for Windows. The level of significance was set to *p* < 0.05 for all tests. Student’s *t*-test was employed for continuous variables, and Chi-square test for categorical ones.

## 3. Results

### 3.1. Demographic and Clinical Characteristics

There were no statistically significant differences in age, sex, and education level between schizophrenic and depressed patients. The clinical characteristics of the patient samples are given in detail in Table 1. The two depressed patients’ subgroups (e.g., bipolar and unipolar) did not differ significantly in their demographic or clinical variables; see Table 2.

### 3.2. Voxel-Based Morphometry Analysis

The gray matter volumes of the two patient groups failed to demonstrate any significant differences when the effects of age, sex, and TIV were accounted for and a stringent statistical threshold of *p* < 0.05 after FWE correction was applied.

### 3.3. Task Related Data Analysis

**Figure 1:**
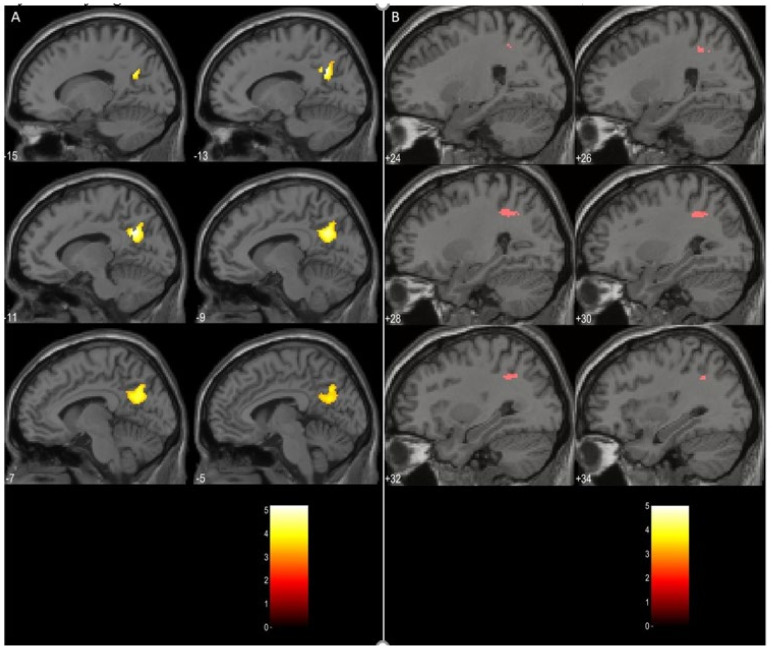
Clusters of activations significantly higher in schizophrenia (**A**)—left precuneus; (**B**)—right posterior parietal lobule.

The comparison between the schizophrenia and the depression group (schizophrenia > depression) using a *t*-test on the contrasts between the DP and the PS blocks resulted in two significant clusters of activations on both cluster and peak level. The first one was localized in the left precuneus extending to the left posterior cingulate gyrus with a cluster size of 376 voxels and a level of significance *p* = 0.034, peak MNI coordinates [−12, −60, 30]. The second cluster, with a size of 72 voxels, encompassed regions of the right superior parietal lobule, and right angular gyrus, *p* = 0.023, peak MNI coordinates [30, −50, 36]. An illustration of these results is given in Figure 1. The opposite comparison (depression > schizophrenia) did not yield any significant clusters.

### 3.4. Effective Connectivity Analysis

#### 3.4.1. Effective Connectivity in the Sample

One sample *t*-test was employed to identify the connections that were significantly different from zero in the whole sample, i.e., both groups. As can be seen in Table 3, all eight nodes had some significant connections, but the most frequently involved ones were precuneus, anterior insula, hippocampus, and orbitofrontal cortex, with three to five such connections. The other three regions—planum temporale, thalamus, and middle frontal gyrus—had only two significant connections with the other nodes. In addition, each of the nodes except for the thalamus demonstrated significant self-inhibitory connections.

#### 3.4.2. Effective Connectivity in the Schizophrenia Group

In the SCH group, the connections that were found to be significantly different from zero included mainly the angular gyrus, anterior insula, and planum temporale. Significant self-inhibitory connections presented the following nodes: precuneus, angular gyrus, planum temporale, and thalamus. These results are given in detail in Table 4.

#### 3.4.3. Effective Connectivity in the Depressed Group

The connections that were identified in the DEP group involved primarily the anterior insula, orbitofrontal cortex, and hippocampus. The self-inhibitory connections were significantly different from zero for the above-mentioned three regions, the middle frontal gyrus, and the angular gyrus as well. The coupling strengths are detailed in Table 5.

#### 3.4.4. Differences between Schizophrenic and Depressed Patients

**Figure 2:**
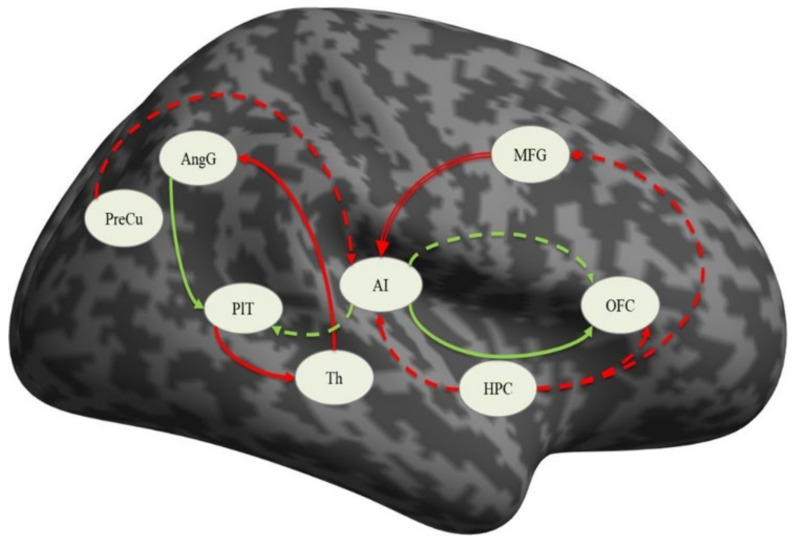
Connections significantly different from zero: solid line—schizophrenia, dashed line—in depression, green—excitatory, red—inhibitory, double red line—significantly different between the groups. PreCu—precuneus, HPC—hippocampus, AI—anterior insula, AngG—angular gyrus, OFC—orbitofrontal cortex, PlT—planum temporale, Th—thalamus, MFG—middle frontal gyrus.

In order to explore the differences between the two groups, independent samples *t*-tests comparing the mean connectivity strengths were performed. The coupling strengths of the connection from the middle frontal gyrus to the anterior insula demonstrated significant difference between the two groups (*p* = 0.041), with the depressed patients having positive mean values (0.054 ± 0.300) but not significantly different from zero (see Table 5) and the schizophrenia patients having negative mean values (−0.112 ± 0.257) that were significantly different from zero (see Table 4). An illustration of these results is presented in Figure 2.

## 4. Discussion

The results of the present study on schizophrenic and depressed patients can be summarized as follows: (i) there were no significant structural differences between the groups in terms of gray matter volumes on a whole-brain voxel-by-voxel comparison; (ii) the SCH group demonstrated significantly more activation in the left precuneus and left posterior cingulate gyrus, as well as the right superior parietal lobule and angular gyrus, during the processing of the paranoid items of the von Zerssen scale when contrasted with the depression items; (iii) the connection from the middle frontal gyrus to the anterior insula was the only significantly different in the direct comparison between the groups, although several other connections at group level seemed to be different as well. The significance of these findings will be discussed in the following lines.

Structural changes as evident in GM volume reductions of different brain regions have been found in schizophrenia and in depression when compared to healthy controls [28,29,30]. However, studies directly comparing the two groups are scarce. In recent years, there has been an increasing number of articles identifying the fronto-temporal regions, insula, and thalamus as more impaired in schizophrenia than in bipolar disorder [31]. On the other hand, the study by Shao et al. did not find significant GM differences between schizophrenia and depression [32]. Thus, the negative results of our study concerning the VBM analysis may be due to the heterogeneity of the depression sample which included both bipolar and unipolar patients.

The results of the task-related data analysis implicated mainly the role of the left PreCu, left PCC, and right AngG. The PCC is a central node in the brain default mode network (DMN) and has strong metabolic activity and strong structural connectivity to multiple brain regions, indicating that it plays a role as a cortical hub [33]. Along with the precuneus, it is considered to be involved in autobiographical memory processing [34]. Structural and functional disturbances in the PCC and PreCu occur in a number of neurological and psychiatric conditions, including neurodegenerative diseases [35], autism spectrum disorder [36], attention deficit hyperactivity disorder [37], and schizophrenia [38].

It is considered that the left PreCu participates, along with the left prefrontal cortex, in the recollection of episodic memories [39], notably those referring to the self [40]. It is also activated when a third-person versus first-person point of view is taken [41]. Along with the superior frontal gyrus and orbitofrontal cortex, the PreCu is activated as individuals render decisions that require behaving out of empathy and forgiveness [42]. Thus, we can speculate that the increased activation of this region in the SCH group during the processing of the Paranoid-Specific items in contrast to the Depression-Specific items is compatible with the hypothesis of stronger involvement of their autobiographical memory.

The right AngG belongs to the inferior parietal lobule and is part of the DMN [43]. The AngG serves as a bridge, where interconnected sensory input is merged and integrated to understand and give context to events, exploit mental representations, redirect attention to the specific information, and focus on solving relevant problems. It is also involved in social cognition and semantic processing as well as memory retrieval [44]. In schizophrenia, where aberrant modulation/activation of the right AngG has been observed [45], it was also correlated with reverse asymmetry in this area [46]. The more significant involvement of this region in schizophrenic patients is most likely related to the specificity of the task e.g., processing of the relevant Paranoid Specific items.

Our analysis of the resting-state fMRI data has demonstrated that in patients suffering from paranoid schizophrenia, significant effective connectivity i.e., causal interaction in terms of excitatory influence, is exerted by the AI on the OFC and by the AngG on the PlT, whereas inhibitory influences are exerted by the MFG on the AI, by the Th (anterior nuclei) on the AngG, and by PlT on the Th. In the patients suffering from a depressive episode, significant excitatory influence is exerted by AI to OFC and to PlT, while inhibitory influences are exerted by PreC and HPC to AI, by HPC to AI, by HPC to OFC, and by HPC to MFG.

However, the comparison between the two groups resulted in one significant connection: the inhibitory influence of the MFG on the AI, which was significantly different from zero only in the SCH group, thereby indicating impaired connectivity between the frontal cortex and the insular cortex. This particular area of the MFG is a part of the Dorsolateral Prefrontal Cortex (DLPFC) and is implicated in the pathophysiology of several neuropsychiatric illnesses [47]. It is among the most recently developed regions of the human brain in terms of evolution, and its maturation continues up until adulthood. The functions that are linked to the DLPFC include sensory feedback, retention in short-term memory, and motor signaling [48]. In addition, the DLPFC is engaged in the decision-making process, including moral decisions as well as risk evaluation [49]. This region is known to be involved in executive functions—cognitive processes such as working memory, cognitive resilience [50], and long-term planning [51]. It is hypothesized that the DLPFC may also be engaged in the act of deception and lying, which is assumed to inhibit the natural propensity to say the truth [52].

The DLPFC dysfunction model of cognitive deficits, behavioral disorganization, and global dysfunction in schizophrenia [53] is supported by the present research. In addition, the identified effects of the connectivity to the anterior insula offer new insights into how dorsolateral prefrontal cortex dysfunction may contribute to the impairment of cognitive functions, behavioral abnormalities, and functional disability observed in people suffering from schizophrenia. We propose a pathophysiological model in which cognitive impairment is present due to the inability to recruit and sustain an organized network between the frontal and insular cortex [53].

In a previous study conducted by our team, Kandilarova et al. [54], we found that depressed patients had a significant reduction in the strength of the right-sided connection from the AI to the MFG, as well as a significant excitatory connection between the amygdala and the anterior insula compared to healthy controls. Because both the Salience Network and the ventral Frontoparietal Network have nodes located in the anterior insular cortex [55], and because of the fact that some authors accept this high degree of correlation between the two networks as evidence that it is only one network [56], we have proposed that our findings add to this evidence by showing the directionality of this disrupted connectivity, namely from the insular cortex to the DLPFC in depressive episodes. However, the connection from the MFG to the AI in this previous study was found not to be significantly different from zero in both healthy controls and depressed patients, which is the case in the present study as well.

The findings of our current study might suggest that the connectivity from DLPFC to the anterior insula can be interpreted as evidence for the presence of an aberrant network that leads to behavioral abnormalities, the manifestation of which depends on the direction of influence. The reduced effective connectivity from the AI to the DLPFC is manifested as depressive symptoms, and the inhibitory effect from the DLPFC to the AI is reflected in the paranoid symptoms of schizophrenia. This suggests that the two psychiatric conditions share a common neural network that is disrupted, but the clinical features depend on the direction of the inhibition and the followed mechanisms from these connectivity disruptions.

In our previous study [54], we speculated that a disruption of the influence of the AI on the Default Mode Network and the Executive Network (as was the case with our sample of depressed patients) may lead to a prevalence of hyperactivity of the DMN. In the current study, we can suggest that the observed inhibitory connection from the DLPFC to AI in patients with schizophrenia might interfere with the balancing function of the insula of the dynamic switch between the DMN. Indirect evidence for that might be the observed activation of both PreCu and PCC (involved in the DMN) in patients with schizophrenia during the task-related fMRI session of the current study.

In conclusion, we can state that the results from our study support the translational cross-validation of the clinical psychological assessment (von Zerssen’s Paranoid-Depressive Scale) by means of functional MRI, where the blocks of visual stimuli represent contrasting items from the clinical scales. At this stage, we can confirm not only the sensitivity of the method (its ability to differentiate healthy controls from patients) but also its specificity (distinction between different psychopathological conditions—in the case of our study, paranoid syndrome in schizophrenia vs. depressive syndrome in MDD or BPD). This methodology can potentially promote the subsequent re-validation of psychiatric classifications and assessment methods based on more reliable evidence-based neurobiological markers.

Moreover, the results from the task-related analysis (residual activations in the Precuneus, the Posterior Cingulate Cortex and the Angular Gyrus) and the disrupted resting state connectivity from the Dorsolateral Prefrontal Cortex (Middle Frontal Gyrus) to the Anterior Insular Cortex observed in the SCH group indicate the involvement of neural networks such as the Salience Network and the Default Mode Network and their abnormal interactions with each other in schizophrenia etiopathogenesis. The Salience Network (SN) is rooted in the dorsal anterior cingulate cortex (dACC) and AI and has predominant limbic and subcortical features. Converging data from structural and functional brain imaging suggest a critical role in the pathophysiology of schizophrenia for both the AI [57] and the DLPFC [58,59]. The abnormal effective connectivity from DLPFC to AI and the involvement of the PreCu as a significant region that activates during task-related fMRI, which we observed in the PS group, suggest that cognitive dysfunction and behavioral disorganization in patients with schizophrenia result from paralimbic-multimodal brain network disintegration rather than focal dysfunction alone. The inhibitory fronto-insular connection leads to disruption of the salience processing and executive systems due to the insular dysfunction as a result of its failure as a dynamic switch between the DMN and SN.

The advanced study performed in this paradigm along with independent replications to follow bring the potential for a fundamental reorganization of psychiatric taxonomy based on the nomothetic networks approach [60]. This approach is constituted by the convergence of multiple measures—molecular, neuroimaging, and phenomenological—to converge on and predict new classes of clinical diagnosis and respective prognosis. It can also be incorporated into the diagnostic process and inform pharmacological therapy by means of multivariate analysis [61].

Additionally, our results point to some interesting and new therapeutic applications that have the ability to modulate SN and thereby DMN function, which seem to be impaired in people suffering from schizophrenia. For example, repetitive transcranial magnetic (rTMS) and direct current stimulation (tDCS) are modern advanced therapeutic methods that have the potential to modulate the network plasticity of the brain [62] and are also promising non-invasive physical interventions. However, the anterior insula is inaccessible for stimulation due to its localization outside the range of rTMS or tDCS. The results of our present study suggest that the anterior insula may still be indirectly modulated by the identified abnormal connectivity of DLPFC to AI by focusing on the frontal cortex, the localization of which makes it accessible for stimulation. The comprehensive approach to improve the dysfunctional SN and DMN observed in patients suffering from schizophrenia is likely to include a combination of pharmacological therapy to restore the aberrant plasticity of brain networks, along with targeted neurostimulation to affect network reorganization. However, this approach and the exact mechanisms that navigate the abnormal interactions between the SN and DMN are not fully understood and need further investigation.

The limitations of our study include the heterogeneity of the sample and the innovative design of our paradigm, which leads to difficulty in attempting to correlate the findings with other similar studies. These limitations could be addressed by extending translational neuroimaging research using a similar approach to the detection of the functional MRI substrate corresponding with the clinical self-assessment tools in replication protocols across independent centers. In order to have more precise results in the future, we need to focus on integrating the knowledge gained not only by single modalities of MRI but also by comparing and integrating the results from task-based fMRI with the residual whole-brain activations observed during the resting-state fMRI, as well as the data from the structural MRI.

## Figures and Tables

**Table 1:** Demographic and clinical characteristics of all participants.

	Schizophrenia Patients (*n* = 25)	Depressed Patients (*n* = 26)	Statistical Significance
**Age (mean ± SD)**	38.8 ± 13.5	41 ± 11.4	0.434 ^a^
**Sex (M/F)**	13/12	9/17	0.210 ^b^
**Education (years)**	13.4 ± 3	13.6 ± 3.3	0.567 ^a^
**Age at onset (years)**	26 ± 9.2	29.6 ± 10.3	0.173 ^a^
**Illness duration (months) **	150 ± 115	139 ± 92	0.885 ^a^
**Episode duration (weeks)**	20.3 ± 28.4	12.6 ± 16	0.141 ^a^

SD—Standard Deviation, ^a^ Independent samples *t*-test, ^b^ χ^2^—test, *p* < 0.05.

**Table 2:** Demographic and clinical characteristics of the two depression subgroups.

	MDD Patients (*n* = 10)	BD Patients (*n* = 16)	Statistical Significance
**Age (mean ± SD)**	37.5 ± 9.9	43.1 ± 12.1	0.286 ^a^
**Sex (M/F)**	4/6	5/11	0.648 ^b^
**Education (years ± SD)**	16 ± 3.7	12.6 ± 2.6	0.113 ^a^
**MADRS score (mean ± SD)**	27.4 ± 4.7	30.4 ± 6.7	0.357 ^a^
**Age at onset (years)**	27.2 ± 6.4	31 ± 12	0.522 ^a^
**Illness duration (months)**	129.2 ± 98.5	144.3 ± 91.3	0.803 ^a^
**Episode duration (weeks)**	10.4 ± 11.2	13.7 ± 18.5	0.490 ^a^

SD—Standard Deviation, ^a^ Independent samples *t*-test, ^b^ χ^2^—test, MADRS—Montgomery–Åsberg Depression Rating Scale, *p* < 0.05, *N*—number of patients, MDD—major depressive disorder, BD—bipolar disorder.

**Table 3:** Connections significantly different from zero in the whole sample.

Connections	Mean	SD	^a^ Significance
**PreCu ⸧**	−0.133	0.290	0.002
**OFC** **→ PreCu**	0.094	0.334	0.027
**HPC ⸧**	−0.091	0.240	0.009
**PreCu** **→ AI**	−0.151	0.328	0.002
**HPC** **→ AI**	−0.128	0.354	0.013
**AI ⸧**	−0.159	0.226	0.000 **
**PreCu** **→ AngG**	0.154	0.420	0.013
**AngG⸧**	−0.160	0.301	0.000 **
**Th** **→ AngG**	−0.117	0.348	0.021
**HPC** **→ OFC**	−0.120	0.316	0.010
**AI** **→ OFC**	0.186	0.335	0.000 **
**OFC⸧**	−0.086	0.293	0.041
**AI** **→ PlT**	0.152	0.292	0.001
**AngG** **→ PlT**	0.088	0.293	0.015
**PlT⸧**	−0.182	0.216	0.000 **
**HPC** **→ Th**	0.080	0.276	0.045
**HPC** **→ MFG**	−0.180	0.333	0.000 **
**Th** **→ MFG**	−0.118	0.345	0.019
**MFG ⸧**	−0.220	0.271	0.000 **

SD—Standard Deviation, ^a^ One-sample *t*-test *p* < 0.05, ** *p* < 0.001, ⸧—self-inhibitory connection, PreCu—precuneus, HPC—hippocampus, AI—anterior insula, AngG—angular gyrus, OFC—orbitofrontal cortex, PlT—planum temporale, Th—thalamus, MFG—middle frontal gyrus.

**Table 4:** Connections significantly different from zero in the schizophrenia group.

Connections	Mean	SD	^a^ Significance
**PreCu ⸧**	−0.156	0.263	0.008
**AI ⸧**	−0.108	0.207	0.017
**MFG** **→ AI**	−0.112	0.257	0.043
**AngG⸧**	−0.161	0.300	0.015
**Th** **→ AngG**	−0.199	0.337	0.011
**AI** **→ OFC**	0.169	0.256	0.004
**AngG** **→ PlT**	0.120	0.265	0.037
**PlT⸧**	−0.214	0.230	0.000 **
**PlT** **→ Th**	−0.156	0.324	0.035
**Th ⸧**	−0.258	0.328	0.000 **

SD—Standard Deviation, ^a^ One sample *t*-test *p* < 0.05, ** *p* < 0.001, ⸧—self-inhibitory connection, PreCu—precuneus, HPC—hippocampus, AI—anterior insula, AngG—angular gyrus, OFC—orbitofrontal cortex, PlT—planum temporale, Th—thalamus, MFG—middle frontal gyrus.

**Table 5:** Connections significantly different from zero in the depressed group.

Connections	Mean	SD	^a^ Significance
**HPC ⸧**	−0.145	0.270	0.011
**PreCu** **→ AI**	−0.200	0.300	0.002
**HPC** **→ AI**	−0.198	0.292	0.002
**AI ⸧**	−0.207	0.236	0.000 **
**AngG⸧**	−0.161	0.310	0.014
**HPC** **→ OFC**	−0.173	0.289	0.005
**AI** **→ OFC**	0.202	0.400	0.016
**OFC⸧**	−0.140	0.241	0.007
**AI** **→ PlT**	0.208	0.311	0.002
**PlT⸧**	−0.154	0.204	0.001
**HPC** **→ MFG**	−0.255	0.357	0.001
**MFG ⸧**	−0.186	0.209	0.000 **

SD—Standard Deviation, ^a^ One sample *t*-test *p* < 0.05, ** *p* < 0.001, ⸧—self-inhibitory connection, PreCu—precuneus, HPC—hippocampus, AI—anterior insula, AngG—angular gyrus, OFC—orbitofrontal cortex, PlT—planum temporale, Th—thalamus, MFG—middle frontal gyrus.

## Data Availability

Data is available upon request.
